# Testing for sexually transmitted infection: who and where? A data linkage study using population and provider data in the Rotterdam area, the Netherlands

**DOI:** 10.1093/fampra/cmad079

**Published:** 2023-08-11

**Authors:** Denise E Twisk, Abraham Meima, Jan Hendrik Richardus, Hannelore M Götz

**Affiliations:** Department of Public Health, Erasmus MC, University Medical Center Rotterdam, Rotterdam, The Netherlands; Department of Public Health, Municipal Public Health Service Rotterdam-Rijnmond, Rotterdam, The Netherlands; Department Research and Business Intelligence, Municipality of Rotterdam, Rotterdam, The Netherlands; Department of Public Health, Municipal Public Health Service Rotterdam-Rijnmond, Rotterdam, The Netherlands; Department Research and Business Intelligence, Municipality of Rotterdam, Rotterdam, The Netherlands; Department of Public Health, Erasmus MC, University Medical Center Rotterdam, Rotterdam, The Netherlands; Department of Public Health, Municipal Public Health Service Rotterdam-Rijnmond, Rotterdam, The Netherlands; Department of Public Health, Erasmus MC, University Medical Center Rotterdam, Rotterdam, The Netherlands; Department of Public Health, Municipal Public Health Service Rotterdam-Rijnmond, Rotterdam, The Netherlands; Centre for Infectious Disease Control, National Institute for Public Health and the Environment (RIVM), Bilthoven, The Netherlands

**Keywords:** demographics, Netherlands, population, registries, sexually transmitted infections, STI clinic, testing, general practitioners

## Abstract

**Background:**

In the Netherlands, insight into sexually transmitted infection (STI) testing and characteristics of those tested by general practitioners (GPs) and sexual health centres (SHC) is limited. This is partly due to lacking registration of socio-demographics at GPs. We aimed to fill this gap by linking different registers.

**Methods:**

Individual STI testing data of GPs and SHC were linked to population register data (aged ≥15 years, Rotterdam area, 2015–2019). We reported population-specific STI positivity, proportion STI tested, and GP-SHC testing rate comparison using negative binomial generalised additive models. Factors associated with STI testing were determined by the provider using logistic regression analyses with generalised estimating equations.

**Results:**

The proportion of STI tested was 2.8% for all residents and up to 9.8% for younger and defined migrant groups. STI positivity differed greatly by subgroup and provider (3.0–35.3%). Overall, GPs performed 3 times more STI tests than the SHC. The smallest difference in GP-SHC testing rate was for 20–24-year-olds (SHC key group). Younger age, non-western migratory background, lower household income, living more urbanised, and closer to a testing site were associated with STI testing by either GP or SHC. GPs and SHC partly test different groups: GPs test women and lower-educated more often, the SHC men and middle/higher educated.

**Conclusions:**

This study highlights GPs’ important role in STI testing. The GPs’ role in the prevention, diagnosis, and treatment of STIs needs continued support and strengthening. Inter-professional exchange and collaboration between GP and SHC is warranted to reach vulnerable groups.

**Key messages**

- Nearly 3% of the general population was tested for a sexually transmitted infection (STI) (2015–2019).
- Proportion of STI tested was up to 9.8% for younger and defined migrant groups.
- General practitioner (GP) test for STIs 3 times more people than the sexual health centre.
- GPs tend to test women and lower-educated people more often.
- Collaboration between GP and sexual health centres is warranted to reach vulnerable groups.

## Background

Testing followed by adequate treatment and partner notification is a key control strategy for sexually transmitted infections (STI). In the Netherlands, general practitioners (GPs) are important providers of STI testing and treatment due to their accessibility and gatekeeper role to secondary healthcare.^1,2^ GPs perform STI tests upon clients’ request or may initiate testing themselves. GPs are advised to perform STI tests based on symptoms or HIV indicator conditions, as well as proactively test key groups such as men who have sex with men, individuals with multiple recent sex partners, and people with a non-western migratory background.^3^ STI consultations at the GP are covered by mandatory health insurance, but laboratory tests may incur costs. Most individuals request an STI test at their GP, and the remainders usually visit a sexual health centre (SHC).^4^ The SHC is an additional free-of-charge service for key groups with the highest STI risk, such as those with STI-related symptoms, having been notified for an STI, men who have sex with men, aged ≤ 24 years, and with a non-western migratory background.^5^ Specialists in hospitals and online testing services have a limited role in STI care in the Netherlands.^4,6,7^

While GPs are significant providers of STI testing, comprehensive information on the characteristics of those tested is only available for SHC clients. Of all SHC attendees in the Netherlands, 23% is non-Western.^8^ The proportion non-western SHC attendees differs largely between SHCs, ranging from 11% to 38%,^9^ depending on the population living in the area. It is unknown how many people with a non-western background visit their GP for an STI test because standard registration of socio-demographics are limited to sex and age.^10^ Also, information on performed STI consultations and tests are not uniformly registered in GP electronic medical records. Two Dutch studies determined the migratory background of GP clients for STI-related consultations; one by linking a national representative sentinel GP network database to the population register,^11^ and another by using questionnaires.^12^ Woestenberg et al. concluded that GPs were generally consulted more often by people with a migratory background compared to native Dutch,^11^ but Trienekens et al. found no difference at all.^12^

The aim of this study is to gain more insight into the background characteristics of people tested for STIs by linking GP and SHC laboratory data to population register data of the greater Rotterdam area. Because we expected differences in test-seeking behaviour by clients and test offering by providers, we also compared GP, and SHC-tested individuals. Information from this study may improve the provision of adequate care by enhancing local STI testing services.

## Methods

This cross-sectional data linkage study was conducted in the greater Rotterdam area of the Netherlands, harbouring broadly 1.3 million residents within 15 municipalities, and covering 367 general practices and one SHC.^13^ The SHC is conveniently situated with public transport stops in the city centre of Rotterdam.

### Data sources, determinants, and outcomes

Non-public population microdata available at the Statistics Netherlands underlie this study (hereafter: population database). All registered residents aged ≥ 15 years living in the study area between 2015 and 2019 were included. The population database was based on data available on January 1st of each year, and included on individual level the determinants: sex, date of birth (age), migratory background based on individual’s and parents’ country of birth, migrants’ generation, highest education qualification, and distance to the closest general practice from home address. Level of education was imputed for < 60 years old, above 60 years missingness was assumed not at random due to absence of national registration. We used multiple imputations via chained equations using 10 iterations of 5 multiple imputations. At 4-digit postal code level of residential location, the database included the determinants: degree of urbanisation and median income per household as indicator for area socio-economic status. In addition, we linked straight-line distance from the postal code centroid to the SHC.^14^

The outcomes in this study were based on STI testing data. We used laboratory data of chlamydia, gonorrhoea, and HIV diagnostic tests performed by GP and SHC from 1/1/2015 to 31/12/2019. All anatomical locations were used, and HIV tests for antenatal screening were excluded. For each study year, we reported dichotomously whether an individual was tested at least once and whether this individual had a positive STI test. GP data were obtained from one laboratory that operates and performs diagnostics for general practices. Depending on the municipality, the laboratory performed diagnostics for 12–100% of all general practices (“GP data coverage”), with a median coverage of 88% (IQR: 60–100%). SHC data were collected from the only SHC in the study area and had a 100% coverage.

### Data linkage

The records in the population database included a unique secured pseudonymised identifier based on citizen service number. This identifier was used to link GP and SHC laboratory STI test data to the population database. The identifier in the GP data was also based on the citizen service number, resulting in a 98% match with the population database. At the SHC, a citizen service number is not registered, and therefore, the identifier was based on a combination of gender, date of birth, and postal code. An identifier was only assigned to SHC clients who had a unique match in the population register. In total, 88% of the SHC clients was provided with an identifier and could be matched to the population database. A comparison of SHC registered characteristics for SHC clients with and without a match is presented in **Supplementary Table 1**.

### Statistical analysis

Data from one of the 15 municipalities in our study area were excluded for analyses, as GP data coverage was considered too low (12%) for reliable estimates. The GP data coverage of the remaining 14 municipalities was between 60% and 100% (median of 90%). First, we described the socio-demographics and postal area level determinants of individuals tested and not tested for an STI. For tested individuals also, the STI positivity (number of diagnoses divided by those tested) was reported. Subsequently, we calculated mean STI testing rates (number of tested individuals per 1,000 residents) at the GP and SHC over the studied years. GP and SHC testing rates were compared using generalised additive models with a negative binomial distribution calculating rate ratios (RR) and their 95% confidence intervals (CI). In these models, SHC was used as a reference, and the log of the total number of residents as offset. We corrected the number of tested individuals for incompleteness. The number of tested individuals by SHC was corrected with 100/88, considering the 88% match between SHC and population data. The number of GP-tested individuals was corrected by 100 divided by the municipality-specific GP laboratory data coverage. Last, to quantify the association between determinants and STI testing by the GP and SHC, we performed logistic regression with generalised estimating equations to account for multiple records per person over the included years. All available determinants had an univariable association with a *P*-value < 0.05 (**Supplementary Table 2**), and were included in the multivariable regression model. No determinants had to be excluded due to multicollinearity; all determinants had a variance inflation factor below 3, with a mean-variance inflation factor of 1.48. The regression analyses were performed without coverage correction. Analyses were conducted with a pooled outcome variable (any chlamydia, gonorrhoea, or HIV). The analyses using generalised additive models and the imputation were performed in R version 3.6.3, and the regression analysis with generalised estimating equations in STATA 16.1. The statistical significance level was set at a *P*-value < 0.05.

## Results

### Characteristics tested and not tested

Per year (2015–2019), approximately 1 million residents aged ≥ 15 years were included. Characteristics of the general population and the STI-tested population are presented in **Table 1**. No difference in characteristics of the general population over the years was observed. Compared to nontested individuals, tested individuals were more often women, younger, non-Dutch, second-generation migrants, and middle-level educated. In addition, they lived in areas with a higher urbanisation level, a lower household income, and nearer to SHC and GP. In total, 2.8% of the general population was tested for an STI, but it was higher among, for example, 20–34-year olds (6.8%) and people with a non-western background (4.7%), especially for Dutch Antilleans (9.8%), Cape Verdeans (7.3%), Sub-Saharan Africans (5.5%), and Surinamese (5.3%). The highest positivity rate was found for 15–19-year olds (30.5%) and the lowest positivity for ages above 35 years (**Table 1**).

**Table 1.** Characteristics of the general population and the population tested for sexually transmitted infections, ≥15 years (2015–2019).

	General population	Not tested^a^	Tested^a^	% positive
	No (%)	No (%)	No (%; row%)	
**Total**	5107921 (100%)	4966797 (100%)	141124 (100%; 2.8%)	15.4%
2015	1005596 (19.7%)	978806 (19.7%)	26790 (19.0%; 2.7%)	15.3%
2016	1012665 (19.8%)	984897 (19.8%)	27768 (19.7%; 2.7%)	15.1%
2017	1021033 (20.0%)	992858 (20.0%)	28175 (20.0%; 2.8%)	15.4%
2018	1029952 (20.2%)	1000968 (20.2%)	28984 (20.5%; 2.8%)	15.6%
2019	1038675 (20.3%)	1009268 (20.3%)	29407 (20.8%; 2.8%)	15.4%
Mean	1021583	993359	28225 (-; 2.8%)	15.3%
Individual				
**Sex**				
Men	2490618 (48.8%)	2433921 (49.0%)	56697 (40.2%; 2.3%)	18.7%
Women	2617303 (51.2%)	2532876 (51.0%)	84427 (59.8%; 3.2%)	13.1%
**Age (years)**				
15–19	350154 (6.9%)	337865 (6.8%)	12289 (8.7%; 3.5%)	30.5%
20–24	407977 (8.0%)	372957 (7.5%)	35020 (24.8%; 8.6%)	21.7%
25–29	445054 (8.7%)	414395 (8.3%)	30659 (21.7%; 6.9%)	15.0%
30–34	423086 (8.3%)	401952 (8.1%)	21134 (15.0%; 5.0%)	11.2%
35–39	396052 (7.8%)	382217 (7.7%)	13835 (9.8%; 3.5%)	9.2%
40–44	398681 (7.8%)	389089 (7.8%)	9592 (6.8%; 2.4%)	7.6%
45–49	440438 (8.6%)	433095 (8.7%)	7343 (5.2%; 1.7%)	7.4%
50–54	435597 (8.5%)	430580 (8.7%)	5017 (3.6%; 1.2%)	8.2%
55–59	404668 (7.9%)	401567 (8.1%)	3101 (2.2%; 0.8%)	7.2%
60–64	358974 (7.0%)	357368 (7.2%)	1606 (1.1%; 0.4%)	7.1%
≥65	1047240 (20.5%)	1045712 (21.1%)	1528 (1.1%; 0.1%)	4.6%
**Migratory background**				
Native Dutch	3260384 (63.8%)	3195373 (64.3%)	65011 (46.1%; 2.0%)	14.2%
Other Western	374454 (7.3%)	363884 (7.3%)	10570 (7.5%; 2.8%)	13.9%
Dutch Antillean	132989 (2.6%)	119958 (2.4%)	13031 (9.2%; 9.8%)	21.2%
Surinamese	305053 (6.0%)	288777 (5.8%)	16276 (11.5%; 5.3%)	16.8%
Turkish	260436 (5.1%)	254279 (5.1%)	6157 (4.4%; 2.4%)	13.0%
Moroccan	189405 (3.7%)	183346 (3.7%)	6059 (4.3%; 3.2%)	15.2%
Other non-Western	266458 (5.2%)	256318 (5.2%)	10140 (7.2%; 3.8%)	13.8%
Sub-Saharan African^b^	57714 (1.1%)	54534 (1.1%)	3180 (2.3%; 5.5%)	13.6%
Cape Verdean	80156 (1.6%)	74323 (1.5%)	5833 (4.1%; 7.3%)	22.1%
Middle and Eastern European	180872 (3.5%)	176005 (3.5%)	4867 (3.4%; 2.7%)	12.6%
**Migratory background** ^c^ **by generation**				
Western (without native Dutch)				
First generation	321663 (57.9%)	313594 (58.1%)	8069 (52.3%; 2.5%)	12.4%
Second generation	233663 (42.1%)	226295 (41.9%)	7368 (47.7%; 3.2%)	14.7%
Non-western				
First generation	838454 (64.9%)	809170 (65.7%)	29284 (48.3%; 3.5%)	13.4%
Second generation	453757 (35.1%)	422365 (34.3%)	31392 (51.7%; 6.9%)	20.5%
**Migratory background** ^c^ **by age**				
Western				
<25 years	489965 (12.8%)	461855 (12.4%)	28110 (34.9%; 5.7%)	21.0%
≥25 years	3325745 (87.2%)	3273407 (87.6%%)	52338 (65.1%; 1.6%)	10.4%
Non-western				
<25 years	268166 (20.8%)	248967 (20.2%)	19199 (31.6%; 7.2%)	28.4%
≥25 years	1024045 (79.2%)	982568 (79.8%)	41477 (68.4%; 4.1%)	11.8%
**Education level** ^d^				
Low	1107656 (34.4%)	1070929 (34.6%)	36727 (28.7%; 3.3%)	20.1%
Middle	1294141 (40.1%)	1232518 (39.8%)	61623 (48.2%; 4.8%)	16.3%
High	822707 (25.5%)	793251 (25.6%)	29456 (23.0%; 3.6%)	10.5%
Missing	1883417	1870099	13318	
**Education level (imputed)** ^d^ ^,^ ^e^				
Low	1420610 (33.0%)	1379793 (33.1%)	40817 (29.1%; 2.9%)	19.0%
Middle	1724767 (40.0%)	1658306 (39.8%)	66461 (47.4%; 3.9%)	15.8%
High	1162096 (27.0%)	1129146 (27.1%)	32950 (23.5%; 2.8%)	10.3%
Missing	800448	799552	696	
Area				
**Degree of urbanisation**				
Very high (≥2,500 addresses/km^2^)	2414666 (47.3%)	2318044 (46.7%)	96622 (68.5%; 4.0%)	15.8%
High (1,500–2,500 addresses/km^2^)	1548797 (30.3%)	1518668 (30.6%)	30129 (21.4%; 1.9%)	15.0%
Moderate (500–1,000 addresses/km^2^)	690718 (13.5%)	681669 (13.7%)	9049 (6.4%; 1.3%)	14.0%
Low (500–1,000 addresses/km^2^)	295389 (5.8%)	291570 (5.9%)	3819 (2.7%; 1.3%)	12.1%
Rural (<500 addresses/km^2^)	156723 (3.1%)	155246 (3.1%)	1477 (1.0%; 0.9%)	12.7%
Missing	1628	1600	28	
**Median household income**				
Highest (>€36,600)	1155750 (22.6%)	1138770 (22.9%)	16980 (12.0%; 1.5%)	12.8%
Upper middle (€28,400–€36,600)	74093 (1.5%)	71867 (1.4%)	2226 (1.6%; 3.0%)	14.6%
Middle (€22,200–€28,400)	1944899 (38.1%)	1900053 (38.3%)	44846 (31.8%; 2.3%)	15.0%
Lower middle (€16,800–€22,200)	1868444 (36.6%)	1794686 (36.1%)	73758 (52.3%; 3.9%)	16.2%
Lowest (<€16,800)	62792 (1.2%)	59510 (1.2%)	3282 (2.3%; 5.2%)	16.5%
Missing	1943	1911	32	
**Distance to closest general practice (in km)** ^f^				
<1	3864777 (75.9%)	3746876 (75.6%)	117901 (84.5%; 3.1%)	15.5%
1–2	1187840 (23.3%)	1166789 (23.6%)	21051 (15.1%; 1.8%)	14.2%
>3	41333 (0.8%)	40797 (0.8%)	536 (0.4%; 1.3%)	14.0%
Missing	13971	12335	1636	
**Distance to SHC (in km)**				
<5	1832795 (35.9%)	1752623 (35.3%)	80172 (56.8%; 4.4%)	15.8%
5–10	1955923 (38.3%)	1913886 (38.5%)	42037 (29.8%; 2.1%)	14.6%
>10	1317575 (25.8%)	1298688 (26.2%)	18887 (13.4%; 1.4%)	15.1%
Missing	1628	1600	28	

*No*, number; *SHC*, sexual health centre; *km*, kilometre.

Data presented as No. and column percentages, unless otherwise indicated. The number of residents tested for an STI is not corrected for data incompleteness.

^a^Chlamydia, gonorrhoea or HIV. (Not) tested by a general practitioner and/or sexual health centre.

^b^Without Cape Verdean.

^c^The code was Western if at least one parent was born in another country in Europe (excluding Turkey), North America, Oceania, Indonesia or Japan. The code was non-Western when at least one parent was born in a country in Africa, Latin America or Asia (excluding Indonesia and Japan) or Turkey. First generation included people who were born abroad; second generation included people who were born in the Netherlands.

^d^The International Standard Classification of Education was used as basis. Low: no education, elementary school, pre-vocational secondary education, senior general secondary education (first 3 out of 5 years), pre-university education (first 3 out of 6 years), secondary vocational education level 1. Middle: senior general secondary education (last 2 out of 5 years), pre-university education (last 3 out of 6 years), secondary vocational education level 2–4. High: university of applied sciences, university.

^e^Multiple imputation via chained equations using ten iterations of 5 multiple imputations. Only imputed for <60 years old, above 60 years missingness was assumed not at random due to absence of national registration.

^f^Based on address of residential location. Other area characteristics are based on the 4-digit postal code of residential location.

### Comparing testing rates by GP and sexual health centre

GPs tested around 3 times more individuals compared to the SHC (RR: 3.09, 95%CI: 3.06–3.12, **Table 2**), with a corrected total of 121,856 versus 39,443 tested individuals in the studied years. A small proportion (3.0%) of the tested population was tested by both providers. The GP-SHC ratio differed per subpopulation, but all subpopulations were tested more often by GPs than by the SHC (**Table 2**). The smallest difference in GP-SHC testing rate was observed for the age group 20–24 years (RR: 1.28, 95%CI: 1.23–1.33). The GP contribution was greater for women (RR: 4.32, 95%CI: 4.28–4.37) compared to men (RR: 2.05, 95%CI: 2.00–2.09), for people with a migratory background (e.g. RR for Turks: 4.10, 95%CI: 3.96–4.23) compared to native Dutch, and for low-educated people (RR: 3.70, 95%CI: 3.64–3.75) compared to high (RR: 2.99, 95%CI: 2.93–3.05) or middle level educated people (RR: 2.55, 95%CI: 2.55–2.60). Based on area characteristics, the relative number of tests by GP was higher in less urbanised areas, and in areas where people live further away from SHC and GP. A less clear trend was observed for household income, due to the RR for upper-middle household income. The STI positivity ranged from 3.0% for ≥ 65-year olds to 27.0% for 15–19-year olds at the GP, and from 14.7% for ≥ 65-year-olds to 35.3% for 15–19-year olds at the SHC (**Table 2**).

**Table 2.** Mean testing rate for sexually transmitted infections, positivity, and comparison between general practitioner and sexual health centre per subpopulation, ≥15 years (2015–2019).

	GP		SHC		STI testing rate GP vs. SHC
	Mean STI testing rate^a^ (n/1,000)	% positive	Mean STI testing rate (n/1,000)	% positive	RR (95% CI)^b^
**Total**	23.9	12.3%	7.7	24.5%	3.09 (3.06-3.12)
2015	22.2	12.3%	8.3	22.6%	2.67 (2.64-2.70)
2016	23.3	12.1%	8.1	23.1%	2.87 (2.83-2.90)
2017	23.4	11.8%	8.2	25.5%	2.89 (2.86-2.92)
2018	24.9	12.6%	7.3	25.3%	3.42 (3.39-3.46)
2019	25.4	12.5%	6.8	26.1%	3.72 (3.69-3.76)
Individual					
**Sex**					
Men	17.6	14.9%	8.6	25.9%	2.05 (2.00-2.09)
Women	29.8	10.8%	6.9	22.8%	4.32 (4.28-4.37)
**Age (years)**					
15–19	27.0	27.0%	14.3	35.3%	1.89 (1.81-1.97)
20–24	56.4	19.3%	44.1	23.6%	1.28 (1.23-1.33)
25–29	61.9	12.5%	16.6	23.2%	3.74 (3.67-3.80)
30–34	48.1	9.4%	8.2	21.5%	5.90 (5.82-5.99)
35–39	34.4	7.4%	4.8	21.8%	7.10 (6.99-7.21)
40–44	24.1	6.2%	2.9	19.6%	8.27 (8.13-8.41)
45–49	16.7	5.8%	2.0	20.3%	8.33 (8.17-8.49)
50–54	11.2	6.0%	1.7	22.7%	6.53 (6.35-6.70)
55–59	7.4	4.7%	1.1	24.3%	6.60 (6.37-6.82)
60–64	4.3	5.0%	0.6	21.1%	6.69 (6.37-7.00)
≥65	1.4	3.0%	0.2	14.7%	6.25 (5.94-6.56)
**Migratory background**					
Native Dutch	16.9	11.1%	5.9	22.9%	2.88 (2.84-2.92)
Other Western	23.9	10.9%	7.7	22.1%	3.09 (3.01-3.17)
Dutch Antillean	87.2	17.7%	26.0	31.5%	3.36 (3.27-3.45)
Surinamese	47.0	13.8%	14.0	25.9%	3.35 (3.27-3.43)
Turkish	21.4	10.2%	5.2	24.2%	4.10 (3.96-4.23)
Moroccan	28.1	12.1%	8.1	25.7%	3.48 (3.35-3.61)
Other non-Western	40.9	10.2%	13.2	24.3%	3.09 (3.02-3.17)
Sub-Saharan African^c^	47.7	11.4%	14.8	21.4%	3.22 (3.05-3.40)
Cape Verdean	64.3	18.9%	19.5	31.5%	3.29 (3.16-3.42)
Middle and Eastern European	24.3	9.8%	6.3	23.1%	3.83 (3.68-3.98)
**Migratory background** ^d^ **by age**					
Western					
15–24 years	37.6	18.5%	29.4	23.2%	1.28 (1.23-1.33)
≥25 years	15.0	8.2%	2.7	22.2%	5.51 (5.46-5.57)
Non-western					
15–24 years	52.4	25.5%	32.0	31.1%	1.64 (1.57-1.70)
≥25 years	38.4	9.8%	7.2	22.1%	5.33 (5.27-5.39)
**Education level** ^e^					
Low	29.9	16.7%	8.1	32.3%	3.70 (3.64-3.75)
Middle	39.3	13.2%	15.4	23.4%	2.55 (2.51-2.60)
High	30.5	7.6%	10.2	19.3%	2.99 (2.93-3.05)
**Education level (imputed)** ^e^ ^,^ ^f^					
Low	26.1	15.7%	6.8	31.7%	3.85 (3.79-3.91)
Middle	32.2	12.7%	12.0	23.3%	2.68 (2.64-2.72)
High	24.5	7.3%	7.7	19.6%	3.17 (3.11-3.23)
Area					
**Degree of urbanisation**					
Very high (≥2,500 addresses/km2)	33.2	12.3%	12.4	24.5%	2.68 (2.64-2.71)
High (1,500–2,500 addresses/km2)	18.1	12.4%	4.3	25.1%	4.17 (4.10-4.23)
Moderate (500–1,000 addresses/km2)	12.4	11.9%	2.7	23.5%	4.63 (4.51-4.74)
Low (500–1,000 addresses/km2)	12.4	10.5%	2.3	21.1%	5.46 (5.27-5.65)
Rural (<500 addresses/km2)	9.5	11.4%	1.7	19.1%	5.60 (5.30-5.89)
**Median household income**					
Highest (>€36﻿,600)	12.6	10.3%	3.6	21.9%	3.53 (3.44-3.61)
Upper middle (€28﻿,400–€36﻿,600)	21.2	9.8%	12.2	22.8%	1.74 (1.55-1.93)
Middle (€22﻿,200–€28﻿,400)	20.8	12.2%	6.0	24.2%	3.49 (3.43-3.54)
Lower middle (€16﻿,800–€22﻿,200)	33.4	12.8%	11.7	25.2%	2.86 (2.82-2.90)
Lowest (<€16﻿,800)	44.4	13.4%	15.6	23.8%	2.85 (2.68-3.02)
**Distance to closest general practice (in km)** ^g^					
<1	26.0	12.4%	8.7	24.6%	2.98 (2.95-3.02)
1–3	16.6	11.8%	4.1	23.5%	4.04 (3.96-4.11)
>3	13.0	11.6%	2.5	26.6%	5.12 (4.65-5.60)
**Distance to SHC (in km)**					
<5	35.3	12.1%	14.5	24.4%	2.43 (2.39-2.47)
5–10	20.2	12.2%	4.7	24.4%	4.26 (4.20-4.32)
>10	13.3	13.0%	2.7	25.3%	4.99 (4.90-5.07)

*CI*, confidence interval; *GP*, general practitioner; *km*, kilometre; *No*, number; RR, rate ratio; *SHC*, sexual health centre; *STI*, sexually transmitted infection.

^a^Based on at least one STI test (chlamydia, gonorrhoea or HIV test). Testing rates are corrected for data incompleteness; the number of tests by SHC was corrected with 1/0.88, considering the 88% match between SHC and population data. The number of tests by the GP was corrected by 1/coverage per municipality.

^b^Comparison of STI testing rate, with SHC as reference.

^c^Without Cape Verdean.

^d^The code was Western if at least one parent was born in another country in Europe (excluding Turkey), North America, Oceania, Indonesia or Japan. The code was non-Western when at least one parent was born in a country in Africa, Latin America or Asia (excluding Indonesia and Japan) or Turkey. First generation included people who were born abroad; second generation included people who were born in the Netherlands.

^e^The International Standard Classification of Education was used as basis. For classification see Table 1.

^f^Multiple imputation via chained equations using ten iterations of 5 multiple imputations. Only imputed for < 60 years old, above 60 years missingness was assumed not at random due to absence of national registration.

^g^Based on address of residential location. Other area characteristics are based on the 4-digit postal code of residential location.

### Determinants associated with being tested

Because the STI testing rate was markedly lowest among people aged > 60 years, we restricted our regression analysis to 15–60-year olds (**Table 3**). Women were more often tested at GP (OR: 1.88, 95%CI: 1.85–1.91), while men were more often tested at the SHC (OR women: 0.85, 95%CI: 0.83–0.88). Overall, 15–19-year-olds and ages over 24 years were tested less often compared to 20–24-year olds (OR ranged from 0.11 to 0.83). Whereas the odds of being tested at the GP was comparable with the SHC for 15–19-year olds, the odds declined much stronger at the SHC beyond 24 years. People with a Dutch Antillean (OR: 2.50, 95%CI: 2.43–2.56), Cape Verdean (OR: 2.08, 95%CI: 2.00–2.16), Surinamese (OR: 1.64, 95%CI: 1.60–1.68), and Sub-Saharan African (OR: 1.29, 95%CI: 1.23–1.35) background were tested more often than native Dutch, while for other groups it was comparable or less often. Overall and at the GP, higher-educated people were tested less often compared to those with low education (overall: OR = 0.88; GP: OR = 0.80). This was not the case at the SHC, where middle (OR: 1.42, 95%CI: 1.37–1.47) and higher educated (OR: 1.24, 95%CI: 1.19–1.29) were tested more often compared to low educated. Generally, the larger the distance to SHC, the smaller the odds of being tested (5–10 km: OR = 0.80; >10 km: OR = 0.65). A less clear trend was observed for distance to the GP. People living in very highly urbanised areas were tested more often by both GP and SHC. No clear association was observed for area-specific household income.

**Table 3.** Determinants associated with testing for sexually transmitted infections^a^ by a general practitioner and/or sexual health centre in residents aged 15–60 years (2015–2019).

	Overall^b^	GP	SHC
	aOR (95% CI)^c^	aOR (95% CI)^c^	aOR (95% CI)^c^
Individual			
**Sex**			
Men	REF	REF	REF
Women	1.55 (1.53–1.58) 1.58)	1.88 (1.85–1.91)	0.85 (0.83–0.88)
**Age (years)**			
15–19	0.46 (0.45–0.47)	0.50 (0.48–0.51)	0.51 (0.49–0.53)
20–24	REF	REF	REF
25–29	0.83 (0.81–0.84)	1.16 (1.14–1.18)	0.38 (0.37–0.40)
30–34	0.63 (0.62–0.64)	0.94 (0.92–0.97)	0.21 (0.20–0.22)
35–39	0.47 (0.46–0.48)	0.72 (0.70–0.74)	0.14 (0.13–0.15)
40–44	0.33 (0.32–0.34)	0.51 (0.49–0.52)	0.09 (0.08–0.09)
45–49	0.23 (0.23–0.24)	0.35 (0.34–0.37)	0.06 (0.06–0.07)
50–54	0.16 (0.15–0.16)	0.23 (0.22–0.24)	0.05 (0.05–0.06)
55–59	0.11 (0.11–0.12)	0.16 (0.15–0.17)	0.04 (0.03–0.04)
**Migratory background**			
Native Dutch	REF	REF	REF
Other Western	1.00 (0.97–1.03)	1.02 (0.99–1.06)	0.91 (0.86-0.96)
Dutch Antillean	2.50 (2.43–2.56)	2.70 (2.62–2.78)	1.86 (1.76-1.97)
Surinamese	1.64 (1.60–1.68)	1.72 (1.68–1.77)	1.39 (1.32–1.46)
Turkish	0.57 (0.55–0.59)	0.63 (0.61–0.66)	0.40 (0.37–0.43)
Moroccan	0.73 (0.70–0.76)	0.80 (0.77–0.83)	0.56 (0.52–0.60)
Other non-Western	0.93 (0.90–0.95)	0.96 (0.93–0.99)	0.84 (0.79–0.89)
Sub-Saharan African^d^	1.29 (1.23–1.35)	1.34 (1.27–1.41)	1.12 (1.01–1.24) ▲
Cape Verdean	2.08 (2.00–2.16)	2.21 (2.12–2.30)	1.70 (1.57–1.84)
Middle and Eastern European	0.61 (0.59–0.64)	0.67 (0.64–0.69)	0.49 (0.45–0.54)
**Education level** ^e^			
Low	REF	REF	REF
Middle	1.06 (1.05–1.08)	0.97 (0.96–0.99)	1.42 (1.37–1.47)
High	0.88 (0.86–0.90)	0.80 (0.79–0.82)	1.24 (1.19–1.29)
Area			
**Degree of urbanisation**			
Very high (≥2,500 addresses/km^2^)	REF	REF	REF
High (1,500–2,500 addresses/km^2^)	0.79 (0.78–0.81)	0.83 (0.81–0.85)	0.70 (0.67–0.74)
Moderate (500–1,000 addresses/km^2^)	0.63 (0.60–0.65)	0.66 (0.64–0.69)	0.54 (0.50–0.58)
Low (500–1,000 addresses/km^2^)	0.67 (0.64–0.71)	0.75 (0.71–0.79)	0.48 (0.43–0.53)
Rural (<500 addresses/km^2^)	0.49 (0.45–0.53)	0.53 (0.49–0.57)	0.36 (0.30–0.44)
**Median household income**			
Highest (>€36﻿,600)	REF	REF	REF
Upper middle (€28﻿,400– €36﻿,600)	1.24 (1.18–1.31)	1.22 (1.14–1.30)	1.17 (1.06–1.28)
Middle (€22﻿,200 – €28﻿,400)	1.17 (1.14–1.20)	1.20 (1.17–1.23)	1.08 (1.02–1.13)
Lower middle (€16﻿,800–€22﻿,200)	1.12 (1.09–1.15)	1.21 (1.17–1.25)	0.91 (0.86–0.97)
Lowest (<€16﻿,800)	1.33 (1.26–1.40)	1.50 (1.42–1.59)	0.97 (0.87–1.07) ■
**Distance to closest general practice (in km)** ^f^			
<1	REF	REF	REF
1–3	0.91 (0.89–0.93)	0.92 (0.90–0.94)	0.89 (0.85–0.92)
>3	1.17 (1.05–1.31)	1.19 (1.05–1.34)	1.09 (0.83–1.43) ■
**Distance to SHC (in km)**			
<5	REF	REF	REF
5–10	0.80 (0.79–0.82)	0.91 (0.89–0.93)	0.56 (0.54–0.59)
>10	0.65 (0.63–0.67)	0.76 (0.74–0.78)	0.41 (0.39–0.44)

*CI*, confidence interval; *GP*, general practitioner; *aOR*, adjusted odds ratio; *No*, number; *REF*, reference; *SHC*, sexual health centre.

^a^Based on at least one STI test (chlamydia, gonorrhoea or HIV test); 15-60-years-olds (917,131 unique persons with 3,690,479 records).

^b^Tested by a general practitioner and/or sexual health centre.

^c^ *P* < 0.01 unless otherwise indicated: ▲ *P* < 0.05, ■ not significant.

^d^Without Cape Verdean.

^e^Imputed level of education. For classification, see Table 1.

^f^Based on address of residential location. Other area characteristics are based on the 4-digit postal code of residential location.

## Discussion

This study linked laboratory STI testing data of GPs and SHC with the Dutch population register to gain insight into the socio-demographics of STI-tested individuals. Nearly 3% of all people were tested for an STI at the GP or SHC, but markedly higher for 20–34-year olds and defined migrant groups (up to 9.8%). GPs generally test for STI 3 times more often than the SHC. Smaller differences in GP-SHC testing rate were observed for SHC key groups like people under 25 years. With the exception of sex and education level, the same individual and area factors were associated with STI testing at the GP or SHC.

This study provides unique information on the STI-tested population in the Netherlands by combining individual-level data from the population register with GP and SHC data. Approximately 3% of the general population were tested for an STI at the GP or SHC, which is comparable to the combined percentages of the estimated STI test consultations at the GP (in 2019: 2.1%; 364,500 consultations among 17,407,585 Dutch residents) and at the SHC (in 2019: 0.9%; 150,782/17,407,585).^7^ We observed large differences in proportion tested and testing rate between subpopulations, with the highest rates for Dutch Antilleans. This was also found by Woestenberg et al., but not by Trienekens et al., who studied GP clients’ migratory background based on estimates^11^ and questionnaires,^12^ with limitations such as selection bias. Compared to these studies, our register-based method has a lower risk of bias, is more objective, highly applicable, and relatively easy to perform behind a desk.

We consider more detailed data on STI testing at the GP necessary for surveillance and control. Previous studies estimated that 70% of all STI/HIV consultations occur at GPs in the Netherlands,^15,16^ but this varies between regions.^17^ This is confirmed in our study for the Rotterdam area. That GPs perform most STI consultations is in line with their accessibility and gatekeeper role to secondary healthcare. People are likely to contact their GP first when noticing any symptoms and/or being unaware of having had the risk of an STI. Other studies showed that GP clients more often reported symptoms than SHC clients (43% vs. 29%),^7,12^ and that symptoms were more often reported as reason for testing at the GP.^6^

Contrary to the GP, SHCs are free-of-charge and considered as additional care. SHC accessibility is limited by strict triage and capacity by governmental finances.^5,18,19^ The latter is reflected by the increasing contribution of the GP over the studied years because SHC finances are unchanged since 2015. The effect of SHC triage policy is, for example, reflected in the higher SHC contribution by young people because < 25-year olds are prioritised. Although people with a non-western background are also prioritised at the SHC, age appears to be more important; testing rates at the SHC hardly differ between western and non-western migrants younger than 25 years. SHC clients are most likely aware of their risk for an STI, are notified by a sex partner, or prefer the SHC above their GP for reasons such as perceived expertise and fear of stigma by visiting a GP in their own neighbourhood. Also, the relatively anonymous and free-of-charge nature of the SHC may be preferable for some groups such as younger people, as people may face out-of-pocket costs when testing at the GP due to health insurance requirements. On the other hand, others may prefer unseen testing by the GP (i.e. a GP visit could be for something other than an STI). For instance, it is known that in Muslim communities cultural sensitives and taboos related to sexuality may prevent individuals from accessing sexual health services and visit their GP instead.^20,21^

At the GP, women, and those with a low education (compared to middle/high education) were more likely to test for an STI. This was not the case at the SHC. Women generally consult healthcare providers more often than men.^22^ On the other hand, men are more likely to be tested at the SHC because men who have sex with men are prioritised and are advised to undergo routine STI testing.^5^ Because men’s test rate is lower and STI positivity higher than for women at the GP, improvements may be possible. GPs are often not aware of clients’ sexual orientation,^23^ but when becoming aware through discussing sexual health (e.g. we “routinely discuss sexual health”) and ask about client’s sexual partners (men, women, or both), they could more often initiate an STI test to male clients who have sex with men, in accordance with national GP guidelines.^3^ It is also noteworthy that people with a different education level seem to navigate to other healthcare providers. In line with a study by Heijne et al., we found that lower educated were more likely to test at the GP.^6^ In addition, low-educated people are slightly underrepresented in the STI tested population (28.7% vs. 34.4% in the general population), despite having a relatively high STI positivity rate. This was also observed among Dutch SHC clients,^24^ but not in combination with GP data. These findings may imply that lower educated are less aware of the SHC or face other barriers accessing them. Moreover, we know that low education level is associated with more risky sexual behaviour and adverse sexual health.^25^ Taken together, this highlights the need to prioritise interventions among lower educated, for example, by outreach testing in the vicinity of where these groups live. Larger distance to testing sites and living in less urbanised areas are also associated with a lower odd of testing, whereas STI-positivity hardly differs between areas. It may therefore be appropriate to start outreach testing in areas further from the SHC and GP. Additionally, one could choose areas with a higher proportion non-western resident as non-western populations have generally a high STI prevalence.^7,26^ The findings of this study could be integrated into continuing medical education for GPs to underscore their role in STI testing. Additionally, the results could serve as background information to emphasise the importance of discussing sexual health with clients and guideline adherence.

### Limitations

There are some limitations to this study. First, we had incomplete GP test data and an incomplete match between SHC data and the population register. In our comparison between GP and SHC, we adjusted for this. We possibly over- or underestimated the testing rates because we did not adjust at a lower level, such as patient characteristics or GP practices. Even with these lower-level correction methods, residual bias may still exist due to unmeasured factors affecting testing. We were not able to adjust in our regression analyses because this was on an individual level. However, as our study included a substantial amount of testing data, we expect that findings would be nearly similar. This is also confirmed by other studies, which found comparable determinants for STI testing.^6,27^ Second, perceived risk, sexual behaviour, reasons to test, and other contextual information are lacking, but these factors often underlie STI testing. Third, the generalisability of our findings may be limited. The greater Rotterdam area, especially the city of Rotterdam, is a high STI prevalence area. Comparison of STI positivity with other countries is challenging due to guidelines and population variations.^28^ Fourth, we used STI testing data, which is not equal to STI-related consultations. Trienekens et al. reported that for 83% of the STI-related consultation an STI test was requested.^12^ Fifth, our pooled STI outcomes are likely driven by chlamydia testing and infections, which is the most recommended STI test by both SHC and GP guidelines, and most frequently diagnosed STI. In practice, combined chlamydia and gonorrhoea tests are usually conducted, and syphilis and/or hepatitis B testing is typically performed in conjunction with HIV testing. We aimed to capture all individuals who were tested for an STI by including 3 of the 5 “big five” STIs. Finally, we observed high testing rates and positivity in some groups, but we were not able to quantify whether the current test rates are sufficient for these groups.

## Conclusions

This study highlights the pivotal role of GPs in STI testing and puts GP-tested clients in perspective to the SHC-tested clients. The available data indicate that GP and SHC basically test similar population groups, with a tendency for GPs to test women and lower-educated people more often. Given the significant role GPs have in STI testing, it is imperative to provide them with continuous medical education on this topic. Inter-professional exchange of experiences and findings, and collaboration between GP and SHC is warranted to develop strategies to reach vulnerable groups such as low-educated individuals. Outreach activities in less urbanised areas, further away from SHC and GP, and in the vicinity of vulnerable groups, may be an appropriate strategy to better reach, for example, low-educated people.

## Supplementary Material

cmad079_suppl_Supplementary_Tables

## Data Availability

Results in this study are based on calculations using non-public microdata from Statistics Netherlands. Statistics Netherlands prohibits data sharing to guarantee the anonymity of the people in its databases. Under certain conditions, microdata are accessible for statistical and scientific research (fees apply). Procedures can be found at https://www.cbs.nl/; for further information, email: microdata@cbs.nl.
