# Supplementary material for: Testing for sexually transmitted infection: who and where? A data linkage study using population and provider data in the Rotterdam area, the Netherlands
Source: Fam Pract. 2023 Aug 11;40(4):599–609. doi: 10.1093/fampra/cmad079 (PMC10667069; doi:10.1093/fampra/cmad079)
Supplement: cmad079_suppl_Supplementary_Tables [file cmad079_suppl_supplementary_tables.pdf]

Supplementary Tables

Supplementary Table 1. Comparison of sexual health centre clients with and without a match to the population register[1] (2015-2019)

	Matched	Not matched	Matched vs. not matched[2]
	No (%)	No (%)	P-value
**Total**	58015 (100.0%)	7691 (100.0%)	
**Sex**			<0.001
Men	35346 (60.9%)	4894 (63.6%)	
Women	22653 (39.0%)	2689 (35.0%)	
Transgender	16 (0.0%)	108 (1.4%)	
**Age**			<0.001
Mean age (SD)	28.67 (10.49)	30.36 (10.85)	
Median age	25	27	
**Non-western migratory background**			<0.001
Yes	22372 (38.6%)	3298 (42.9%)	
No	35643 (61.4%)	4393 (57.1%)	
**Migratory background**			<0.001
Native Dutch	30714 (53.0%)	3461 (45.0%)	
Other Western	4899 (8.4%)	928 (12.1%)	
Dutch Antillean	4859 (8.4%)	608 (7.9%)	
Surinamese	5622 (9.7%)	517 (6.7%)	
Turkish	1761 (3.0%)	197 (2.6%)	
Moroccan	1968 (3.4%)	319 (4.1%)	
Other non-Western	5387 (9.3%)	1090 (14.2%)	
Cape Verdean	1894 (3.3%)	142 (1.8%)	
Middle and Eastern European	881 (1.5%)	425 (5.5%)	
**Education level**			<0.001
Low/medium	25164 (43.4%)	3140 (40.8%)	
High	32055 (55.3%)	4194 (54.5%)	
Other/unknown	796 (1.4%)	357 (4.6%)	
**Triage criterium**			
MSM	19213 (33.1%)	3107 (40.4%)	<0.001
STI-related symptoms	16022 (27.6%)	1704 (22.2%)	<0.001
Notified about STI exposure	12199 (21.0%)	1354 (17.6%)	<0.001
Performing sex work	1464 (2.5%)	796 (10.3%)	<0.001
**STI test and diagnosis**			
Tested for Chlamydia	57519 (99.1%)	7625 (99.1%)	0.977
Chlamydia positive	9277 (16.1%)	1023 (13.4%)	<0.001
Tested for Gonorrhoea	57526 (99.2%)	7625 (99.1%)	0.891
Gonorrhoea positive	4177 (7.3%)	533 (7.0%)	0.391
Tested for HIV	38953 (67.1%)	6004 (78.1%)	<0.001
HIV positive	191 (0.3%)	33 (0.4%)	0.158

No. (%) unless otherwise indicated. Abbreviations: HIV, human immunodeficiency virus; MSM, men who have sex with men; No, number; SD, standard deviation; SHC, sexual health centre; STI, sexually transmitted infection.
[1] Includes all SHC clients and compares SHC registered characteristics. In total 50607/58015 (87.2%) of the match SHC clients lived in the greater Rotterdam area (no age selection).
[2] Based on Chi square test.

Supplementary Table 2. Univariable regression analysis of determinants associated with testing for sexually transmitted infections[1] by a general practitioner and/or sexual health centre in residents aged 15-60-years (2015-2019)

	**Overall**[2] OR (95% CI)[3]	**GP** OR (95% CI)[3]	**SHC** OR (95% CI)[3]
**Individual**			
**Sex**			
Men	REF	REF	REF
Women	1.54 (1.51-1.56)	1.85 (1.82-1.88)	0.87 (0.84-0.89)
**Age (years)**			
15-19	0.42 (0.41-0.43)	0.48 (0.46-0.49)	0.38 (0.37-0.40)
20-24	REF	REF	REF
25-29	0.82 (0.81-0.84)	1.14 (1.12-1.16)	0.40 (0.39-0.41)
30-34	0.60 (0.59-0.61)	0.90 (0.88-0.92)	0.20 (0.19-0.21)
35-39	0.43 (0.41-0.44)	0.65 (0.63-0.67)	0.12 (0.12-0.13)
40-44	0.29 (0.28-0.30)	0.45 (0.44-0.46)	0.07 (0.07-0.08)
45-49	0.20 (0.19-0.21)	0.31 (0.30-0.32)	0.05 (0.05-0.05)
50-54	0.14 (0.13-0.14)	0.21 (0.20-0.22)	0.04 (0.04-0.04)
55-59	0.10 (0.09-0.10)	0.15 (0.14-0.15)	0.03 (0.03-0.03)
**Migratory background**			
Native Dutch	REF	REF	REF
Other Western	1.29 (1.25-1.32)	1.28 (1.24-1.32)	1.29 (1.23-1.36)
Dutch Antillean	3.91 (3.81-4.01)	4.13 (4.01-4.25)	3.20 (3.04-3.37)
Surinamese	2.15 (2.10-2.21)	2.28 (2.22-2.34)	1.83 (1.74-1.91)
Turkish	0.87 (0.84-0.90)	0.95 (0.92-0.99) ▲	0.64 (0.59-0.69)
Moroccan	1.18 (1.14-1.23)	1.25 (1.21-1.30)	0.98 (0.91-1.05) ■
Other non-Western	1.41 (1.37-1.45)	1.44 (1.39-1.48)	1.34 (1.27-1.41)
Sub-Saharan African[4]	1.95 (1.86-2.05)	2.03 (1.92-2.14)	1.68 (1.52-1.84)
Cape Verdean	3.06 (2.95-3.18)	3.24 (3.11-3.37)	2.57 (2.39-2.77)
Middle and Eastern European	0.98 (0.94-1.02) ■	1.06 (1.02-1.11)	0.76 (0.70-0.83)
**Education level**[5]			
Low	REF	REF	REF
Middle	1.27 (1.26-1.29)	1.17 (1.16-1.19)	1.61 (1.56-1.66)
High	0.98 (0.97-1.00) ∆	0.95 (0.93-0.97)	1.06 (1.02-1.10)
**Area**			
**Degree of urbanisation**			
Very high (≥2,500 addresses/km²)	REF	REF	REF
High (1,500–2,500 addresses/km²)	0.55 (0.54-0.56)	0.60 (0.59-0.61)	0.40 (0.39-0.42)
Moderate (500-1,000 addresses/km²)	0.36 (0.35-0.37)	0.40 (0.38-0.41)	0.25 (0.23-0.26)
Low (500-1,000 addresses/km²)	0.37 (0.36-0.39)	0.42 (0.40-0.44)	0.22 (0.20-0.24)
Rural (<500 addresses/km²)	0.28 (0.26-0.30)	0.32 (0.29-0.34)	0.17 (0.15-0.20)
**Median household income**			
Highest (>€36.600)	REF	REF	REF
Upper middle (€28.400 - €36.600)	1.82 (1.73-1.92)	1.56 (1.47-1.66)	2.74 (2.50-3.00)
Middle (€22.200 - €28.400)	1.62 (1.59-1.66)	1.62 (1.58-1.66)	1.69 (1.61-1.77)
Lower middle (€16.800-€22.200)	2.43 (2.38-2.48)	2.34 (2.29-2.40)	2.83 (2.71-2.96)
Lowest (<€16.800)	3.06 (2.92-3.22)	3.02 (2.86-3.19)	3.43 (3.12-3.78)
**Distance to closest general practice (in km)**[6]			
<1	REF	REF	REF
1-3	0.64 (0.63-0.65)	0.67 (0.66-0.68)	0.54 (0.52-0.56)

>3	0.50 (0.45-0.55)	0.53 (0.48-0.60)	0.35 (0.28-0.45)
**Distance to SHC (in km)**			
<5	REF	REF	REF
5-10	0.57 (0.56-0.58)	0.64 (0.63-0.65)	0.39 (0.38-0.40)
>10	0.39 (0.38-0.40)	0.45 (0.44-0.46)	0.23 (0.22-0.24)

Abbreviations: CI, confidence interval; GP, general practitioner; km, kilometre; No, number; OR, odds ratio; REF, reference; SHC, sexual health centre; STI, sexually transmitted infection.

[1] Based on at least one STI test (chlamydia, gonorrhoea or HIV test).

[2] Tested by a general practitioner and/or sexual health centre.

[3] $p<0.01$ unless otherwise indicated: ▲ $p<0.05$, Δ $p<0.1$, ■ not significant.

[4] Without Cape Verdean.

[5] Imputed level of education. Multiple imputation via chained equations using ten iterations of five multiple imputations. The International Standard Classification of Education was used as basis. For classification see Table 1 in the main text.

[6] Based on address of residential location. Other area characteristics are based on the 4-digit postal code of residential location.
